# Current Views and Perspectives on E-Mental Health: An Exploratory Survey Study for Understanding Public Attitudes Toward Internet-Based Psychotherapy in Germany

**DOI:** 10.2196/mental.6375

**Published:** 2017-02-23

**Authors:** Jennifer Apolinário-Hagen, Viktor Vehreschild, Ramez M Alkoudmani

**Affiliations:** ^1^ Institute for Psychology Department of Health Psychology University of Hagen, Faculty of Humanities and Social Sciences Hagen Germany; ^2^ Kulliyyah of Pharmacy Pharmacy Practice Department International Islamic University Malaysia Kuantan, Pahang Malaysia

**Keywords:** telemedicine, mental health, online self-help, attitude to computers, acceptability of health care, patient acceptance of health care, cognitive therapy, stress, psychological, diffusion of innovation

## Abstract

**Background:**

Despite the advanced development of evidence-based psychological treatment services, help-seeking persons with mental health problems often fail to receive appropriate professional help. Internet-delivered psychotherapy has thus been suggested as an efficient strategy to overcome barriers to access mental health care on a large scale. However, previous research indicated poor public acceptability as an issue for the dissemination of Internet-delivered therapies. Currently, little is known about the expectations and attitudes toward Internet-delivered therapies in the general population. This is especially the case for countries such as Germany where electronic mental health (e-mental health) treatment services are planned to be implemented in routine care.

**Objective:**

This pilot study aimed to determine the expectations and attitudes toward Internet-based psychotherapy in the general population in Germany. Furthermore, it aimed to explore the associations between attitudes toward Internet-based therapies and perceived stress.

**Methods:**

To assess public attitudes toward Internet-based psychotherapy, we conducted both Web-based and paper-and-pencil surveys using a self-developed 14-item questionnaire (Cronbach alpha=.89). Psychological distress was measured by employing a visual analogue scale (VAS) and the 20-item German version of the Perceived Stress Questionnaire (PSQ). In addition, we conducted explorative factor analysis (principal axis factor analysis with promax rotation). Spearman’s rank correlations were used to determine the associations between attitudes toward Internet-based therapies and perceived stress.

**Results:**

Descriptive analyses revealed that most respondents (N=1558; female: 78.95%, 1230/1558) indicated being not aware of the existence of Internet-delivered therapies (83.46%, 1141/1367). The average age was 32 years (standard deviation, SD 10.9; range 16-76). Through exploratory factor analysis, we identified 3 dimensions of public attitudes toward Internet-based therapies, which we labeled “usefulness or helpfulness,” “relative advantage or comparability,” and “accessibility or access to health care.” Analyses revealed negative views about Internet-based therapies on most domains, such as perceived helpfulness. The study findings further indicated ambivalent attitudes: Although most respondents agreed to statements on expected improvements in health care (eg, expanded access), we observed low intentions to future use of Internet-delivered therapies in case of mental health problems.

**Conclusions:**

This pilot study showed deficient “e-awareness” and rather negative or ambivalent attitudes toward Internet-delivered therapies in the German-speaking general population. However, research targeting determinants of the large-scale adoption of Internet-based psychotherapy is still in its infancy. Thus, further research is required to explore the “black box” of public attitudes toward Internet-delivered therapies with representative samples, validated measures, and longitudinal survey designs.

## Introduction

### Background

Mental health problems requiring treatment have a high lifetime prevalence of 29.2%, which has increased over the past decades across the world [1]. The incidence of mental health problems across populations and long waiting times for psychotherapy in many regions indicate a demand for innovative effective prevention and treatment strategies in public health. Despite the advanced development of evidence-based treatments for a broad range of mental health problems, still many individuals requiring treatment fail to receive professional help in primary care [2,3]. In addition, stigmatized beliefs about mental illnesses have been identified as global problem for both help-seeking persons as well as for the diffusion of mental health services [4-6]. With respect to limited capacities of health care, the dissemination of Internet-delivered psychological services is suggested as an efficient strategy to improve the access to professional help by overcoming structural or regional barriers [2,3,7,8] and the stigma of seeking help for mental health problems [9,10]. Internet-delivered computerized, electronic mental health (e-mental health) services include the usage of modern digital technologies and new media in, for instance, monitoring, screening, psychoeducation, prevention, health promotion, self-help, counseling, aftercare, and psychotherapy [11]. Concerning treatment delivered through the Internet, controlled trials have confirmed the effectiveness for guided Internet-based cognitive behavior therapy (iCBT) and related approaches for mood and anxiety disorders [12-14], eating disorders [15], coping with chronic somatic conditions [16], and harmful health behavior [17].

However, these promising findings from controlled studies appear to challenge the overall poor uptake of e-mental health services in health care systems worldwide indicating psychological barriers [18-21]. Although clinical studies have identified individual predictors of engagement or disengagement in active treatment conditions [22-25], the evidence base for predictors of help-seeking intentions and using e-mental health treatments in the general population is still scare. An English study by Musiat et al [26] targeting public acceptability of e-mental health treatment services showed that mental health service users endorsed domains such as helpfulness, credibility, convenience of access, personal support, or suitability with preferences and habits as important for decisions to engage with these services [26]. Taken together, most studies targeting the general population in this field directed to low willingness to future use of e-mental health services in case of emotional distress [10,26-29]. Potential reasons for negative expectations and attitudes toward e-mental health include concerns on privacy [28,30,31], communication [9], therapeutic alliance, and unfamiliarity with technology [1]. Facilitators or positive attitudes and perspectives of Internet-based therapies need to be explored.

### Attitudes Toward E-Mental Health and Service Users’ Acceptance

Ajzen [32] defined attitudes as the sum of affective appraisals either positive or negative to a psychological object on attributive dimensions ranging, for example, from harmful to beneficial or helpful. Attitudes toward using self-help are assumed to be affected by individual experiences with mental disorders, self-help services and seeking help in primary care, as well as with perceived control, helplessness, engagement, and self-stigma [33]. There are indications for multidimensional (ambivalent) attitudes in terms of health behavior [32,34]. This appears important for the measurement of attitudes toward health-related topics because attitudes are typically assumed to be located within a unidimensional continuum, that is, positive or negative attitudes, but not both for the same object [34]. In recent years, attitudes toward e-mental health treatments have been mainly investigated among patients [35-37] and health care professionals [38-40]. For instance, the “Attitudes towards Psychological Online Interventions Questionnaire” (APOI) [36] is a validated measure with depressive patients in a German clinical setting. Yet, validated measures targeting public attitudes toward Internet-based treatments outside the context of clinical studies are rare. In addition, research findings from countries with advanced eHealth infrastructure, such as Australia, United States, or Canada [2], are not directly applicable to the assessment of public attitudes toward Internet-based therapies in countries such as Germany due to different stages of e-health implementation into health care. There is thus a need to identify general determinants of e-mental health adoption in the general population.

As framework, the technology acceptance model [41] and its extension, the unified theory of acceptance and use of technology (UTAUT) [42], can help to identify determinants of behavioral intentions to use information technology (IT). The multidisciplinary UTAUT [43,44] is based on 8 models developed in psychology, sociology, human-computer- interaction, and IT acceptance research, including the innovation diffusion theory [45] and the theory of planned behavior (TPB) [46]. Four determinants of behavioral intentions to use IT and moderators of key relationships (eg, gender, age, experience) have been confirmed [43,44]. Determinants include performance expectancy, effort expectancy, social influence, and facilitating conditions. The determinant with the best predictive value for usage intentions called “performance expectancy” consists of perceived usefulness, relative advantage, extrinsic motivation, and outcome expectations. Concerning the assessment of IT in treatment research, perceived usefulness or helpfulness appears particularly relevant. Accordingly, surveys employed perceived helpfulness as an indicator for attitudes and acceptability of e-mental health services [10,47-49].

### Social Influence, Perceived Stress, and Attitude Toward E-Mental Health Treatments

Overall, it remains largely unclear how affective and cognitive information processing affects the formation of attitudes [32]. Studies have shown mediating effects of perceived stress on (face-to-face) help-seeking intentions [50]. Predictors of help seeking and using (face-to-face) mental health services are assumed to include positive attitudes toward seeking professional help, increased levels of perceived psychological distress, as well as lower social support [51]. However, little is known about the associations between perceived stress, help-seeking intentions, and attitudes toward using modern technologies. In addition, explorative studies outside of clinical settings mostly identified inconsistent or weak associations. For instance, a pilot study [52] showed that perceived stress was negatively associated with attitudes to computers among students. Considering the outlined limited evidence base on the role of distress (as control variable) in attitudes toward e-mental health services and help-seeking intentions, further research is urgently required. Moreover, the measurement of ambivalent attitudes and their impact on health-related behavior needs clarification [32]. This is relevant given the discrepancy between promising findings and low impact of e-mental health in public health.

### E-Mental Health in the German Health Care Context

In 2015, 44.5 million persons (63%) of the general German population used the Internet daily, whereas 56.1 million citizens (79.5%) had access to the Internet [53]. The implementation of e-mental health treatments in German public health could help reducing the gap between supply and demand for psychotherapy [3]. Web-based self-help services are accessible for the German public, but professional regulation is a barrier for the dissemination of (therapist-guided) Internet-based therapies. Currently, treatment delivered by health professionals exclusively through the Internet is prohibited in routine care due to the so-called “Fernbehandlungsverbot” in Germany [54]. However, the implementation of Internet-delivered therapies is considered in German public health [3]. Public opinions about e-mental health treatment services have been rarely explored in Germany. A survey with a representative sample of the German general population by Eichenberg et al [55] showed that more than one-third of 2411 respondents indicated using the Internet for mental health advice. Regarding Web-based interventions, the awareness of respondents was very low. This study [55] also demonstrated that most respondents preferred seeking information or help from face-to-face services in case of emotional distress. In line with international research [10,26], specific subpopulations (eg, young adults) indicated being more willing to use e-mental health [55]. However, the study by Eichenberg [55] did not focus on public attitudes toward Internet-based therapies. In addition, data were collected in 2010. Since then, the diffusion of modern technology into everyday life (eg, mobile phone apps) has likely increased the public awareness in Germany. Therefore, it appears reasonable to explore the “status quo” of public attitudes toward e-mental health treatments in Germany again (5 years later).

### Objective

Concerning the intended large-scale implementation of e-mental health treatment services into primary care, this pilot study aimed to determine expectations and attitudes toward Internet-based psychotherapy in the German-speaking general population. Another purpose of this survey was to explore the associations between the attitudes toward Internet-delivered therapies and perceived stress as well as individual differences in attitudes in terms of gender, age, “e-awareness,” and experience with psychotherapy.

## Methods

### Study Design and Setting

A cross-sectional survey using a psychometric observational study design was conducted. This pilot study combined both validated and self-developed self-report measures. All items were provided in German language through both an anonymously conducted Web-based survey and a paper-and-pencil survey. Data from the paper-and-pencil survey were collected between April and August 2015, whereas data from the Web-based survey were collected between June and August 2015 using Google forms. No ethical approval was required. No details about the medical history (eg, diagnosed mental disorders), clinical screenings, or other problematic areas, including identifiable names or region (protection of confidentiality and privacy), were assessed. A convenience sample was obtained using snowballing techniques (nonprobability sampling). Data were based solely on self-reports.

### Participants

Participants older than 16 years were recruited from German-speaking general population through social network sites, such as Facebook, professional networks, and undergraduate psychology courses at different universities across North Rhine Westphalia, Germany. Participation was voluntary. Psychology students could receive credits for their participation. No further incentive was offered.

### Measures and Procedure

Candidates received brief text-based information about this survey, including its objectives and conditions for participation (informed consent). The survey consisted of 3 parts. The first part included sociodemographic questions, experience with psychotherapy, Internet usage, awareness of Internet-based therapies, and a single item on the current stress level. The second part was a self-developed 14-item questionnaire on attitudes toward Internet-based therapies. Due to the novelty of the study subject, the term Internet-based therapy was explained for laypersons prior to attitude assessment. The instruction included information about evidence base (especially depression and anxiety) and current stage of implementation of Internet-delivered therapies in German health care in comparison to other countries. The final part of the survey was the assessment of 20 items on stressful events in the last 4 weeks. The average time for completing the survey amounted to 7 minutes.

### Sociodemographic Variables, Mental Health Care Experience, and E-Awareness

Sociodemographic questions included gender, age, housing situation (alone vs not alone), the area of residence (rural vs urban), educational level, and employment status. Participants were asked to indicate whether they had experience with face-to-face psychotherapy. The frequency of Internet usage was measured as an indicator for familiarity with new media. Participants were asked to indicate whether they ever had heard or read about Internet-based psychotherapies to assess the percentage of e-mental health awareness (“e-awareness”). “E-awareness” was only measured in the Web-based survey.

### Attitudes Toward Internet-Based Psychotherapy

A 14-item self-developed questionnaire was used to explore public attitudes toward Internet-based psychotherapy (Table 2). We developed this brief self-report measure due to the lack of validated instruments on public attitudes or acceptability of e-mental health treatment services. Existing self-report measures were either developed in clinical contexts [36] or not directly applicable to the German public health context [10,26]. Items of the measure were selected based on a literature review that aimed to identify commonly cited statements about relative advantages of e-mental health treatment services for mental health care [2,3,18]. The main findings of this work were published as rapid review [21]. The first set of items was subsequently modified after an expert interview (licensed psychotherapist with a senior level of clinical expertise). The expert interview was used to clarify and discuss the suitability of items. The pilot version of the measure was pretested with 14 persons (both students and laypersons without health care background). Feedback of this pretesting involved clarity of items to improve the face validity of the instrument. The instruction contained a brief description about the delivery mode and most common indications (best evidence base for mild-to-moderate mood and anxiety disorders). It also mentioned that Internet-delivered therapies are available in the Netherlands and that they are also considered for implementation in German primary care.

Participants were asked to indicate their agreement to each of the 14 statements about Internet-delivered psychological treatments on a 5-point rating scale, ranging from 0 (“strongly disagree”) to 4 (“strongly agree”). As shown in Table 2, most items of the measure referred to attitudes in terms of subjective appraisals about statements on proposed benefits of Internet-delivered therapies for persons with mental health problems. Other items reflected expectations about the potential positive impact of Internet-delivered therapies for mental health care. Based on theoretical framework [32,34], we defined attitudes toward Internet-based therapies as sum of negative, neutral, or positive assessments about a psychological object, situation, or setting. As heuristic for the classification of attitudes, we defined threshold or cutoff values in terms of mean and median scores: Values smaller than 1.5 were defined as negative, scores between 1.5 and 2.5 as neutral, and scores greater than 2.5 as positive attitudes toward Internet-based therapies. In addition, we assessed ambivalent attitudes indirectly considering the qualitative coherence or match of positive or negative attitude toward Internet-based therapies across extracted factors and items of the self-developed survey. For instance, despite positive views regarding the helpfulness of Internet-delivered therapies, other aspects such as intentions to future use Internet-based therapy could be assessed negatively at the same time (indicating ambivalent attitudes). The labeling and mapping of items to factors derived through explorative factor analyses were intended to be based on the UTAUT framework [42,43]. Cronbach alpha reliability of the 14-item measure amounted to .89 in this survey.

### Assessment of Stress Perceptions

#### Current Stress Level

A single-item rating scale was used to assess the current subjective stress level. Such visual analogue scales (VAS) were widely used to measure subjective feelings in medical conditions [56]. VAS included a continuous line with 2 endpoints containing only the smallest and maximal value for the subjective assessment. Participants were asked to indicate to what extent they felt stressed at the moment on a VAS ranging from 0 (“not at all”) to 10 (“maximal”).

#### Perceived Stress Questionnaire

The 20-item short version of the Perceived Stress Questionnaire (PSQ-20) [57] was used to assess stress perceptions during the last 4 weeks. Participants were asked to indicate how often the presented statements applied to themselves on a 4-point Likert scale ranging from 1 “almost never” to 4 “usually.” The PSQ-20 consisted of the 4 subscales “worries,” “demands,” “tension,” and “joy” and an overall score. In a German validation study on the PSQ long version, internal consistency was good; Cronbach alpha amounted to .86 for the overall score and ranged between .80 and .85 for the 4 subscales [57].

### Statistical Analyses

Data of respondents with completed attitude measure analyses were considered for analyses. Descriptive analyses were applied to summarize scores of self-report measures, including means, median scores, and standard deviations (SDs). For data reduction purposes, we conducted an exploratory factor analysis (EFA) for the attitude measure using principal axis factoring as extraction method and promax with Kaiser normalization as oblique rotation procedure (κ=4). Oblique rotation was chosen due to the assumption that the items were not independent from each other (attitude definition). The number of extracted factors was derived using the Kaiser-Guttman criterion for eigenvalues greater than 1. Factor loadings smaller than .10 were suppressed. Additionally, the scree plot was examined for characteristic changes in slope. Both Bartlett test of sphericity and Kaiser-Meyer-Olkin (KMO) criterion were used to confirm the suitability of data for the EFA. Regression factor scores were derived. Factor labels were based on both structure and pattern matrices. The mapping was grounded on the UTAUT. Associations between attitudes, stress perceptions, and age were calculated. Spearman rank correlation (ρ coefficient) was used because it was more robust than Pearson correlation coefficient in case of questionable multivariate normal distribution. Additionally, we explored differences in mean (*t* tests) and variance (univariate variance analysis; analysis of variance, ANOVA) to identify differences in e-therapy attitudes based on gender, “e-awareness,” and therapy experience. Internal consistency of the e-therapy attitude measure was assessed using Cronbach alpha. Effect sizes of correlational analyses were classified as small, medium, or large with respect to Cohen criteria [58]. All statistical tests for significance (two-tailed hypotheses with alpha=.05) were performed using SPSS, version 23 (IBM Analytics).

## Results

### Descriptive Analyses

A total of 1559 responses were collected through both a Web-based survey (1456) and a paper-and-pencil survey (103). One respondent indicated being 14 years old and was thus excluded from data analyses. This resulted in a final sample size of 1558 responses. The average age of participants was 32 years (mean 31.6, SD 10.9, median 28 years). Most respondents were females (78.95%, 1230/1558), residing in a German city or urban region (70.86%, 1104/1558), living together with at least one other person or persons in their household (69.51%, 1083/1558), and using the Internet daily (96.92%, 1510/1558). Table 1 shows a summary of sample characteristics differentiated by data collection through Web-based and paper-and-pencil surveys.

**Table 1:** Sample characteristics (N=1558).

Variable		Web-based (n=1455)	Paper-and-pencil (n=103)
**Age**	Mean (SD), years	31.48 (10.72)	31.42 (13.45)
	Range (median), years	16-75 (28)	19-76 (25)
**Gender**	Female, n (%)	1160 79.73)	70 (67.96)
	Male^d^, n (%)	295 (20.27)	33 (32.04)
**Employment status^e^**	Employment, n (%)	697 (47.90)	63 (60.17)
	University student or full-time, n (%)	468 (32.16)	14 (13.59)
	Occupational studies or part-time, n (%)	118 (8.11)	49 (47.57)
	Trainee or pupil (secondary education), n (%)	96 (6.60)	6 (5.83)
	Self-employment, n (%)	93 (6.39)	3 (2.91)
	Unemployment, n (%)	76 (5.22)	2 (1.94)
	Parental leave, n (%)	62 (4.26)	1 (0.97)
	Retirement, n (%)	51 (3.51)	9 (8.74)
	Vocational retaining or rehabilitation, n (%)	39 (2.68)	1 (0.97)
**Education**	No school certificate, n (%)	12 (0.82)	1 (0.97)
	Basic school qualification^a^, n (%)	87 (5.98)	4 (3.88)
	Secondary school (“Mittlere Reife”)^b^, n (%)	387 (26.60)	11 (10.68)
	(%) German “Abitur” or “Fachabitur”^c^, n (%)	613 (42.13)	70 (67.96)
	University degree (Bachelor- or Master level), n (%)	340 (23.37)	16 (15.53)
	Postgraduate or postdoctoral degree, n (%)	16 (1.10)	1 (0.97)
**Region of residence** **Housing situation**	Area in or near a city or urban area, n (%)	1038 (71.34)	66 (64.08)
	Living not alone in the household, n (%)	1011 (69.45)	72 (69.90)
**Experience with psychotherapy^f^**	No, n (%)	547 (37.59)	50 (48.54)
	Yes, as patient	656 (45.09)	39 (37.86)
	Yes, as relative, n (%)	259 (17.80)	14 (13.59)
	Yes, as professional, n (%)	201 (13.81)	5 (4.85)
**Internet usage** **(frequency)**	Daily, n (%)	1423 (97.80)	87 (84.47)
	Several times a week, n (%)	32 (2.20)	10 (9.71)
	Several times a month, n (%)	0 (0)	3 (2.91)
	Rarely or occasionally, n (%)	0 (0)	0 (0)
	Very rare or never, n (%)	0 (0)	3 (2.91)
**E- Awareness** **(Internet-delivered therapies)**	No (not aware), n (%)	1141 (83.46)	Not investigated in the paper-and-pencil survey
	Yes (aware), n (%)	190 (13.97)	
	Not sure, n (%)	35 (2.56)	
	Missing	88 (6.05)	

^a^Basic school qualification=9 school years.

^b^Secondary school (“Mittlere Reife”)=10 years.

^c^German “Abitur” or “Fachabitur”=12-13 years.

^d^One respondent in the Web-based survey reported male as sex, but being “bigender” (commentary section).

^e^Employment status: maximum 2 answers were possible.

^f^Experience with psychotherapy: multiple answers (max. 3 answers for the ”yes” option). A total of 26 of 1558 participants reported experience with psychotherapy in 3 roles (as patient, relative, and professional).

The mean score for the 14-item e-therapy attitude measure amounted to mean 1.79 (SD 0.71; n=1553). Both modes of data collection resulted in comparable mean scores, although it was identified as slightly lower in the paper-and-pencil survey sample (n=103). The mean score for e-therapy attitudes was mean 1.52 (SD 0.59, median 1.5; n=100, n=3 missing) in the paper-and-pencil sample and mean 1.81 (SD 0.59, median 1.79; n=1455) in the Web-based sample. Table 2 summarizes the descriptive analyses for items of the e-therapy attitude measure, the VAS on current stress, and the PSQ-20 on stress perceptions in the last 4 weeks.

**Table 2:** Summary of means, standard deviations, and median for stress and attitude assessments (N=1558).

Variables			Mean (SD)	Median
Stress perceptions				
**PSQ-20 overall score (past 4 weeks)**			52.97 (6.08)	53.33
		PSQ-20 subscale “demands”	54.87 (12.01)	53.33
		PSQ-20 subscale “tension”	50.15 (11.91)	46.67
		PSQ-20 subscale “worries”	48.39 (16.53)	46.67
		PSQ-20 subscale “joy”	57.42 (25.94)	60.00
Current stress level (range: 0 to 10)			5.94 (2.40)	6.5
E-therapy attitudes				
**Overall score attitude assessment (mean score)**			1.79 (0.71)	1.79
		Internet-based therapies are modern and in line with our modern times^a^.	.93 (1.02)	1.0
		Internet-based therapies will replace conventional face-to-face psychotherapy in the future.	2.39 (1.01)	3.0
		Internet-based therapy is better compatible with work and private life than conventional face-to-face therapy.	0.88 (1.12)	.0
		It makes no difference to me whether psychotherapy is conducted through the Internet or in a psychotherapy practice in a clinic.	2.56 (1.16)	3.0
		Internet-based therapies will reach more individuals with mental health problems.	3.07 (0.97)	3.0
		Internet-based therapies can help bridging waiting time for conventional psychotherapy.	3.02 (1.02)	3.0
		Health insurance companies should cover the costs for Internet-based therapies.	1.37 (1.05)	1.0
		Internet-based therapy programs are as effective as conventional face-to-face psychotherapies.	1.13 (1.13)	1.0
		Trust in a therapist can be just as easily built on the Internet as in conventional face-to-face psychotherapy	1.09 (1.06)	1.0
		Regarding therapeutic success, it makes no difference whether contacts with a therapist are provided via the Internet or face-to-face in a psychotherapeutic practice.	1.51 (1.10)	2.0
		Internet-based therapies are an appropriate alternative to conventional face-to-face psychotherapy.	1.71 (1.33)	2.0
		In case of mental health problems, I would attend an Internet-based therapy.	1.04 (1.22)	1.0
		I would prefer an Internet-based therapy to a conventional face-to-face psychotherapy.	2.24 (1.14)	2.0
		Internet-based therapies will reach more patients and help them.	2.18 (0.92)	2.0
	

^a^All items were translated from German language. The rating scale of the e-therapy attitude measure ranged from 0 “strongly disagree” to 4 “strongly agree.” Item 1 refers to expectations and can be interpreted best in connection to other attitudinal items.

As presented in Table 2, most respondents tended to disagree to most of presented statements on suggested advantages of Internet-delivered therapies (ie, 6 items with mean and median scores ≤1.5). The number of items with positive assessment (score≥2.5) and neutral assessments (score between 1.5 and 2.5) was equally distributed (each with 4 statements meeting the criteria).

### Explorative Factor Analysis for the E-Therapy Attitude Measure

The EFA resulted in the extraction of 3 factors, which were labeled as “usefulness or helpfulness” (factor 1, 6 items), “relative advantage or comparability“(factor 2, 5 items), and “e-Accessibility or health care” (factor 3, 3 items).

As shown in Multimedia Appendix 1, we identified significant inter-correlations between the 14 items of the e-attitude measure ranging up to *r*_1558_=.68 (*P*<.001) for items 8 and 9. In contrast to the other items, item 14 was regularly uncorrelated with other items of the measure. The identified significant inter-correlations between most items supported the decision to conduct EFA with oblique rotation instead of using more common orthogonal rotation (ie, varimax). Suitability of data for conducting the EFA with oblique rotation was confirmed given both the results of the Bartlett sphericity test (χ^2^_91_=10420.515, *P*<.001) and the KMO index (.918) for sampling adequacy. Table 3 shows the pattern matrix for the 3-factor-solution EFA. The structure matrix (see Multimedia Appendix 2) and the rationale for labeling factors extracted through the EFA (see Multimedia Appendix 3, [42]) have been presented.

The total explained variance amounted to 63.71% (unrotated sum of square factor solutions with respect to the extraction; “factor 1” with 43.36%, “factor 2” with 13.02%, and “factor 3” with 7.33% of explained variance). The rotated sums of square factor solutions amounted to 40.42% for “factor 1,” 9.02% for “factor 2,” and 3.02% of explained variance for “factor 3.” The rotated sums of squared loadings were as follows: 4.78 (factor 1), 4.70 (factor 2), and 1.50 (factor 3).

Cronbach alpha reliabilities for standardized items were very good for the 14-item measure (14 items, alpha=.88; unstandardized items alpha=.89). Both factors “usefulness or helpfulness” (6 items, alpha=.87 for both standardized and unstandardized items) and “relative advantage“(5 items, alpha=.84 for both standardized and unstandardized items) showed good Cronbach alpha reliability scores (internal consistency). However, for the third factor “e-accessibility,” we identified a poor alpha reliability (3 items, alpha=.30 for unstandardized and alpha=.27 for standardized items). This should be considered because it was no actual factor, but a single item.

Because of the outlined issues for “factor 3,” we calculated a second factor analysis with a 2-factor solution. This 2-factor-solution EFA is presented in Multimedia Appendix 4. Due to a loss of explained variance identified for this 2-factor solution, we decided to focus on the findings of the 3-factor solution in this study. Another reason was that this third factor (item 14) was mostly unrelated to other items of the measure. This indicated another, yet uncovered dimension of e-therapy attitudes. For the calculation of correlations, we used the 3-factor solution.

**Table 3:** Pattern matrix: factor loadings of the exploratory factor analysis (EFA) with promax for the e-therapy attitude measure (Extraction method: principal axis factor analysis; rotation method: promax with Kaiser normalization).

Items of the e-therapy attitude measure	Factors^a^		
	1. Usefulness or helpfulness	2. Relative advantage	3. e-Accessibility health care
7. Health insurance companies should cover the costs for Internet-based therapies.	.882^c^	–.142	.125
1. Internet-based therapies are modern and in line with our modern times.	.833^c^	–.205	+++^b^
3. Internet-based therapy is better compatible with work and private life than conventional face-to-face therapy.	.807^c^	–.181	+++^b^
12. In case of mental health problems, I would attend an Internet-based therapy.	.574^c^	.284	–.121
9. Trust in a therapist can be just as easily built on the Internet as in conventional face-to-face psychotherapy.	.516^c^	.409	–.151
8. Internet-based therapy programs are as effective as conventional face-to-face psychotherapy.	.506^c^	.374	+++^b^
4. It makes no difference to me whether psychotherapy is conducted through the Internet or in a practice in a clinic.	–.303	.855^c^	.109
13. I would prefer an Internet-based therapy to a conventional psychotherapy.	–.113	.854^c^	+++^b^
2. Internet-based therapies will replace conventional face-to-face psychotherapy in the future.	+++^b^	.578^c^	.177
11. Internet-based therapies are an appropriate alternative to conventional face-to-face psychotherapy.	.309	.545^c^	+++^b^
10. Regarding therapeutic success, it is incidental whether contacts with a therapist are provided via the Internet or face-to-face in a practice.	.400	.488^c^	+++^b^
5. Internet-based therapies will reach more people with mental health problems.	+++^b^	.319	.540^c^
6. Internet-based therapies can help bridging waiting time for conventional psychotherapy.	.341	+++^b^	.473^c^
14. Internet-based therapies will reach more patients and help them.	+++^b^	+++^b^	–.111^c^

^a^Factor loadings smaller than .1 were suppressed (+++).

^b^Item rotation converged in 6 iterations.

^c^Mapping of items to factor: bold values indicate that the highest factor loading on a factor.

### Associations Between Perceived Stress and E-Therapy Attitudes

As presented in Table 4, the 3 factors extracted through EFA and overall mean score of the e-therapy attitude measure were all significantly positively correlated with the current stress level (VAS) and the PSQ subscale “joy” (with small effect sizes). Respondents rated Internet-based therapies as more positive if they indicated to feel more stressed at the time of study participation. This was viewed in the context of stress as control variable. However, pleasant experiences or “joy” in last 4 weeks were also slightly associated with positive assessments in the e-therapy attitude measure. Moreover, the factors “usefulness or helpfulness,” “e-accessibility,” and the overall mean score of the e-therapy attitude measure were significantly positively associated with the PSQ-20 overall score (all with small effect sizes). In contrast, the PSQ subscale “worries” correlated significantly negatively with both “relative advantage” and “e-accessibility” (with small effect sizes), but not with the overall mean score of the e-therapy attitude measure. In other words, self-rated worries were associated with tendencies to negative views about the accessibility and relative advantages of Internet-delivered therapies. The only significant (negative) correlation with the PSQ subscale “tension” was identified for the factor “e-accessibility” (albeit with small effect size). No significant correlation was found between the PSQ subscale “demands” and the scores of the e-therapy attitude measure (Table 4).

**Table 4:** Correlation matrix: associations between perceived stress and e-therapy attitudes (N=1558).

Stress variables	e-Therapy attitudes			
	e-Therapy attitudes (mean score)	Usefulness or helpfulness	Relative advantage or comparability	e-Accessibility health care
PSQ overall score	0.055^a^	.053^a^	.046^b^	.060^a^
PSQ demands	–0.019^b^	–.042^b^	–.043^b^	−.035^b^
PSQ worries	–0.023^b^	–.035^b^	–.069^a^	–.115^a^
PSQ tension	–.011^b^	.001^b^	–.047^b^	–.051^a^
PSQ joy	0.079^a^	.078^a^	.135^a^	.168^a^
Current stress (VAS)	0.056^a^	.077^a^	.080^a^	.147^a^

^a^Spearman rank correlation (rho, ρ), significant correlation (*P* ≤.05).

^b^Spearman rank correlation (rho, ρ), not significant correlation (*P*>.05).

#### Correlations With Age

Significant positive correlations were found between age and the mean score of e-therapy attitudes (ρ_1552_=.109; *P*<.001), for factor 1 “usefulness or helpfulness” (ρ_1552_=.108; *P*<.001) and factor 2 “relative advantage or comparability” (ρ_1552_=.097; *P*<.001), but not for factor 3 “e-Accessibility or health care” (ρ_1552_=.006; *P*=.81 not significant). In addition, negative correlations between age with PSQ overall score (ρ_1455_=–.068; *P*=.009) were identified. In contrast, age was correlated neither with any of the 4 PSQ-20 subscales nor with the VAS score on the current stress level (all not significant).

#### Gender Differences

The survey consisted of 1226 females (e-therapy attitudes: mean 1.78; SD 0.70) and 332 males (e-therapy attitudes: mean 1.83; SD 0.74). According to the Levene test, error variances between both gender groups were homogenously distributed (*F*_1551_=.808; *P*=.37, not significant). As the *t* test showed, there were no significant differences in e-therapy attitudes in mean scores between females and males in this survey (*t*_1551_=1.050; *P*=.29, not significant).

#### E-Awareness Differences

About three-quarters of respondents (73.3%, 1141/1558) indicated being not aware of the existence of Internet-delivered therapies (e-therapy attitudes: mean 1.80, SD 0.70, n=1141). In total, 190 respondents stated being aware of Internet-based therapies (e-therapy attitudes: mean 1.87, SD 0.80, n=190) and 36 persons reported being not sure whether they ever heard or read about Internet-delivered therapies (e-therapy attitudes: mean 2.23, SD 0.72, n=36). Although the sample sizes were highly unequal, group comparisons were performed for explorative analyses. As the Levene test showed, the error variances for the mean score of e-therapy attitudes were not homogenously distributed across the 3 e-awareness groups (*F*_3,1451_=2.353; *P*=.07, not significant). The univariate ANOVA revealed no significant between-subjects effect of “e-awareness” on e-therapy attitudes (*F*_1,3_=1.726; *P*=.16, not significant).

#### Experience With Psychotherapy Differences

As the Levene test showed, error variances for the mean score of e-therapy attitudes were homogenously distributed across 7 groups (*F*_7,1447_=1.750; *P*=.09, not significant). Tests of between-subjects effect were undertaken with e-therapy attitudes as dependent variable. Univariate ANOVA revealed no significant effect of previous psychotherapy on e-therapy attitudes (*F*_1,7_=1.421; *P*=.19, not significant).

Taken together, explorative analyses of associations between stress perceptions and attitudes toward Internet-delivered therapies yielded significant findings, albeit with weak effect sizes. In addition, age was found to be correlated with attitudes. Other individual differences in e-therapy attitudes were not identified.

## Discussion

### Principal Findings

This pilot study addressed the question of how Internet-delivered therapies are perceived in the German-speaking population. Another objective of this survey was to explore the associations between attitudes toward Internet-based therapies and perceived stress. Overall, this survey showed ambivalent attitudes. The main findings and implications for future research are discussed in the following sections.

### Summary of Main Findings

#### Public Attitudes Toward Internet-Delivered Psychotherapy

In summary, our study findings indicated ambivalent attitudes toward Internet-based psychotherapy in a sample of 1558 persons from the German-speaking general population. Ambivalent attitudes involved the coemergence of contradicting (both negative and positive) appraisals about Internet-delivered therapies. Analyses revealed predominantly negative views about Internet-delivered therapies. Most respondents disagreed with statements on advocated advantages of Internet-based therapies with respect to subjective norm, effectiveness (outcome expectancy), compatibility, therapeutic support (facilitating factors), and the willingness to the future use. This was in line with previous studies [10,26,59,60] that also identified negative view acceptability and low likelihood of using Internet-based therapies in case of mental health problems. The lowest agreement was found for the statement that Internet-delivered therapies are better compatible with work and life conditions than traditional psychological services. This finding appears surprising because Internet-based therapies are suggested to provide more flexibility in terms of time and location as barriers to access professional help [2,3,18,26].

As noted earlier, the e-therapy attitude measure also identified positive assessments about the proposed advantages of Internet-delivered therapies. Analyses showed positive views about “e-accessibility.” Respondents were rather optimistic about the potential of digital therapies in mental health care to reach more patients and help them through treatment delivered via the Internet. The highest agreement of participants was found for the statement about the expanded public access or reach of Internet-based therapies for people with mental health problems. In addition, respondents endorsed Internet-based therapies as a valid strategy to bridge waiting time of conventional therapies. This optimistic assessment was, however, challenged by other findings. Interestingly, we also identified a low agreement regarding the statement that health insurance should cover the costs for Internet-based therapies. This appeared to contradict the identified positive views on the helpfulness of Internet-based therapies. Thus, these incoherent patterns of ratings could be interpreted as indications for ambivalent public attitudes in this surveyed convenient sample of the German-speaking general population.

Another point to consider was that the labeling and mapping of e-therapy attitudes to factors were associated with uncertainties. This was because of the commonalities of the 3 factors of the e-therapy attitude measure. Data analyses suggested that different dimensions for the assessment of Internet-based therapies were interrelated and partly hard to identify as distinct components of attitudes. Although we tried to achieve match with constructs of the UTAUT [42,43], we were aware that our self-developed 14-item questionnaire had only a loose connection to the original framework. For instance, we understood “perceived usefulness” in a broader sense, which included expectations about both the helpfulness of Internet-delivered treatments for individual adopters and advances in health care in general. Specific adaptions of the UTAUT framework to the measurement of public attitudes toward e-mental health treatment services could be thus the next logic step. Furthermore, the conducted EFA resulted in a 3-factor solution for the e-therapy attitude measure, but the 2-factor solution for the e-attitude measure appeared more plausible given that fact that the third factor was represented by only 1 item. Hence, the e-therapy attitude measure should be tested with a representative sample to make definitive conclusions regarding its structure.

#### Individual Differences in Attitudes Toward Internet-Delivered Psychotherapy

This study showed significant positive associations between perceived stress and e-therapy attitudes. Respondents who reported perceiving more stressful events were also more likely to assess Internet-delivered therapies as beneficial. Several explanations appear plausible for this finding. Perceived stress and the increased need for social support could affect help-seeking intentions; related coping strategies and attitudes toward seeking help were identified in earlier research [33,50]. In addition, the study findings indicated that respondents with higher perceived distress appeared being more open to future use of Internet-delivered treatment services, such as iCBT. A possible reason for this finding is that distressed persons had sought for professional support on the Internet and were thus rather ready to use mental health services in comparison to persons who felt currently less distressed. Another explanation is that perceived stress had not directly affected attitudes, but mediated the relationship between individual motives and intentions to use mental health services, as shown in an earlier study [50]. Given the exploratory nature of this survey and the novelty of the e-therapy attitude measure, we did not conduct mediation analysis. Moreover, it should be also considered that subjective assessments of current stress perceptions could have been biased by affective heuristics and contextual clues [61]. Both the novelty of e-mental health treatment services and the low awareness of Internet-based therapies identified in this convenience sample could have resulted in participants using affective heuristics, biasing their assessments about the usefulness of Internet-delivered therapies toward more neutral or negative views. The issue of low “e-awareness” and rather negative assessments about e-mental health treatment services was also observed in previous researches [26,48]. This assumption was supported by the finding that most respondents of the online sample in this study (83.46%, 1141/1367) reported being definitely not aware of the existence of Internet-delivered therapies prior to their survey participation.

To sum up, the evidence base for public attitudes toward Internet-based therapies is too small to make definite conclusions. Although this survey provided some insights, it had limitations. Hence, further research is needed to determine the role of both perceived stress and “e-awareness” in public attitudes toward seeking help on the Internet in case of mental health problems.

### Limitations

This pilot study has several limitations. Consistent with other studies targeting public attitudes toward e-mental health [10,26,48], we used a self-developed survey that was not validated. In addition, convenience sampling in this study could have been affected by selection bias. This is a common issue in e-mental health research [62]. Nonetheless, sample characteristics of this study were in line with the findings showing that females and well-educated younger persons were the main groups seeking health information on the Web [63,64] and engaging with e-mental health interventions [22]. Moreover, we identified a high percentage of unawareness regarding the existence of Internet-based therapies. The single item on “e-awareness” was also a limitation. Future studies should consider operationalizing “e-awareness” using a multi-item measure with precise descriptions of intervention formats. Comparisons made in the items of attitude measure were also a limitation because conventional therapies might be viewed as “benchmark” and thus Internet-based therapies as inferior [26]. In Germany, Internet-delivered treatments are not covered by health insurances. Additionally, psychotherapists face legal barriers to provide Internet-based treatments. This uncertain legal status could also play a role in forming negative attitudes regarding the effectiveness or appropriateness of Internet-delivered psychotherapy. However, this hypothesis needs further investigation. Additionally, it remains unclear on which basis respondents have built their opinions about Internet-delivered therapies. It can be assumed that subjective definitions of the term “Internet-based therapy” varied broadly. It remains unclear to what extent lacking “e-awareness” has affected the willingness to future using Internet-delivered therapies in case of emotional distress. Moreover, identified associations between attitudes and perceived stress were weak. The theoretical grounding of the correlational research question was also not strong. Thus, for further validation purposes, other constructs than those applied (ie, stress) in this study could be a better choice. A final point to consider is the 3-factor structure of the measure identified through EFA and related psychometric issues. This left some questions open. Taken together, the findings presented in this study should be therefore interpreted with caution.

### Implications

It can be assumed that the ongoing diffusion of e-mental health services into everyday life will affect attitudes toward using these services over the course of the next years. Exploring public attitudes toward e-mental health treatment services on a regular basis is thus recommended throughout different stages of their implementation into German health care. Experience with these innovations is likely to influence public opinions. For instance, an Australian study [65] demonstrated that both laypersons and health professionals were more likely to endorse e-mental health treatments as helpful when they had used them in the past. Psychoeducational information and e-mental health literacy could also improve the acceptability and attitudes toward Internet-based therapies [48,66]. Nonetheless, it should be noted that the role of “e-awareness” in attitudes toward Internet-delivered therapies is still understudied [26,48]. There are indications that lacking e-awareness is an obstacle for assessments, resulting in more negative views about e-mental health services [26]. However, in this study we used a vague definition of Internet-based therapies that might have resulted in an overweight of negative assessments. Participants in this pilot study were asked to assess their views on Internet-based therapies, regardless of medical indication, intervention type, or delivery mode of Internet-based therapies. It is possible that presenting specific types or provision modes of Internet-delivered therapies in the survey instruction could have resulted in type-specific differences in e-therapy attitudes, as previous research suggested [10,26,48]. Therefore, different aspects of Internet-based therapies should be considered in the measurement of attitudes to provide deeper insights into the “black box” of individual determinants underlying the adoption of e-mental health treatments [67]. For this purpose, the psychometric assessment of e-therapy attitudes could be combined with qualitative methods, such as in-depth interviews [7] or focus group discussions [9]. Because qualitative approaches require substantially more resources as well as efforts in comparison to quantitative surveys, mixed methods could be a viable strategy to improve the operationalization of constructs in surveys targeting e-therapy attitudes in nonclinical populations.

The findings of this study directed to options to improve the psychometric assessments of attitudes toward Internet-based therapies. As modification of the study design, descriptions of specific formats of Internet-delivered therapies to determine type-specific preferences should be included in the instruction of the e-therapy attitude measure. Another modification for the e-therapy attitude measure we considered was the employment of additional items reflecting disadvantages, concerns, and psychological barriers. For instance, barriers to seek help such as the stigma of mental illness could be diminished on the Internet [27] besides misunderstandings and false interpretations [68], impersonal communication [59], or concerns about privacy and data security [10,27,30,55]. We integrated these aspects in a revised version of e-attitude measure [69]. Based on the EFA presented in this study, we excluded 2 items with high loading scores on more than 1 factor (ie, items 6 and 10) and added 5 novel statements reflecting data security concerns, benefits of anonymous access, risk of misunderstanding, and unequal accessibility for underprivileged populations. With this revised 17-item measure, we expect to cover more components of e-therapy attitudes as it appeared to be a multidimensional construct.

Finally, despite our broad recruitment strategy we had a highly selected sample consisting mainly of young adults and females. Although more female than male persons seek help for mental problems and attend psychotherapy [51], future studies should aim to explore attitudes of populations underrepresented in most studies, such as young men [70]. Given that Internet-delivered therapies such as iCBT mostly reach similar populations as traditional face-to-face CBT [21,62], expanding the public access to professional help remains a great challenge. Understanding the views and needs of a broad range of potential adopters in public health could be a crucial next step to reach more or hard-to-reach persons from the general population.

### Conclusions

This study revealed mostly negative, ambivalent attitudes toward Internet-delivered therapies with poor “e-awareness” in the German-speaking general population. However, e-mental health research scoping on public attitudes toward e-mental health treatments is still in its infancy. The self-developed measure, marginal e-awareness, and the nonrepresentative sample were limitations of this study. Hence, further research regarding the development and validation of measures is recommended to shed more light into the “black box” of public perceptions of Internet-based therapies. Nonetheless, active participation of both citizens and patients in the development of Internet-based interventions is important, and thus future research should aim to extend options for innovative ways to assess public attitudes toward e-mental health treatment services.

## Appendix

Multimedia Appendix 1

Multimedia Appendix 2

Multimedia Appendix 3

Multimedia Appendix 4

## Abbreviations

ANOVA: analysis of variance
CBT: cognitive behavior therapy
e-awareness: e-mental health awareness
EFA: exploratory factor analysis
e-mental health: electronic mental health
e-therapy attitudes: electronic or Internet-based therapy attitudes
iCBT: Internet-based cognitive behavior therapy
IT: information technology
KMO: Kaiser-Meyer-Olkin (index)
NS: not significant
PAF: principal axis factor analysis
TAM: technology acceptance model
TPB: theory of planned behavior
UTAUT: unified theory of acceptance and use of technology
VAS: visual analogue scale
