# Supplementary material for: Current Views and Perspectives on E-Mental Health: An Exploratory Survey Study for Understanding Public Attitudes Toward Internet-Based Psychotherapy in Germany
Source: JMIR Ment Health. 2017 Feb 23;4(1):e8. doi: 10.2196/mental.6375 (PMC5378055; doi:10.2196/mental.6375)
Supplement: Multimedia Appendix 1 [file mental_v4i1e8_app1.pdf]

Multimedia Appendix 1

Table A1. Summary of Intercorrelations between the Items of the E-Therapy Attitudes Measure (N=1558).

**Item[a]**	**1**	**2**	**3**	**4**	**5**	**6**	**7**	**8**	**9**	**10**	**11**	**12**	**13**	**14**
**1**	1.0	.19**	.59**	.08**	.17**	.28**	.60**	.43**	.43**	.43**	.37**	50**	.20**	.07**
**2**		1.0	.16**	.49**	.44**	.32**	.21**	.36**	.34**	.45**	.45**	.32**	.48**	-.06*
**3**			1.0	.09**	.18**	.28**	.56**	.44**	.45**	.42**	.37**	.49**	.20**	.04
**4**				1.0	.43**	.30**	.15**	.34**	.33**	.42**	.46**	.27**	.66**	-.01
**5**					1.0	.47**	.27**	.29**	.28**	.38**	.41**	.24**	.49**	-.03
**6**						1.0	.46**	.40**	.38**	.39**	.45**	.33**	.39**	-.10**
**7**							1.0	.57**	.58**	.54**	.49**	.55**	.31**	.04
**8**								1.0	.68**	.60**	.56**	.55**	.46**	.00
**9**									1.0	.65**	.56**	.56**	.48**	.03
**10**										1.0	.59**	.54**	.55**	-.03
**11**											1.0	.64**	.60**	-.04
**12**												1.0	.42**	.01
**13**													1.0	-.01
**14**														1.0

Pearson Correlation Coefficients **P*<.05, ** *P*<.01, both two-tailed.

[a] *Items of the e-therapy attitudes measure*: 1. Internet-based therapies are modern and in line with our modern times. 2. Internet-based therapies will replace conventional face-to-face psychotherapy in the future. 3. Internet-based therapy is better compatible with work and private life than conventional face-to-face therapy. 4. It makes no difference to me whether psychotherapy is conducted through the Internet or in a practice in a clinic. 5. Internet-based therapies will reach more people with mental health problems. 6. Internet therapies can help bridging waiting time for conventional psychotherapy. 7. Health insurance companies should cover the costs for Internet-based therapies. 8. Internet-based therapy programs are as effective as conventional face-to-face psychotherapy. 9. Trust in a therapist can be just as easily built on the Internet as in conventional face-to-face psychotherapy. 10. Regarding therapeutic success, it makes no difference whether contacts with a therapist are provided via the internet or face-to-face in a psychotherapeutic practice. 11. Internet therapies are an appropriate alternative to conventional face-to-face psychotherapy. 12. In case of mental health problems, I would attend an Internet-based therapy. 13. I would prefer an Internet-based therapy to a conventional psychotherapy. 14. Internet-based therapies will reach more patients and help them.
