# Supplementary material for: Current Views and Perspectives on E-Mental Health: An Exploratory Survey Study for Understanding Public Attitudes Toward Internet-Based Psychotherapy in Germany
Source: JMIR Ment Health. 2017 Feb 23;4(1):e8. doi: 10.2196/mental.6375 (PMC5378055; doi:10.2196/mental.6375)
Supplement: Multimedia Appendix 2 [file mental_v4i1e8_app2.pdf]

Multimedia Appendix 2

Table A2. Structure matrix. factor loadings of the exploratory factor analysis (EFA) with promax for the e-therapy attitude measure (N = 1558).

Items of the E-therapy attitudes measure	Factors		
	**1.Usefulness or Helpfulness**	**2.Relative Advantage**	**3.E-Accessibility Healthcare**
7. Health insurance companies should cover the costs for Internet-based therapies.	**.820**	.432	.234
9. Trust in a therapist can be just as easily built on the Internet as in conventional face-to-face psychotherapy	**.733**	.659	.102
12. In case of mental health problems, I would attend an Internet-based therapy.	**.721**	.580	+++
1. Internet-based therapies are modern and in line with our modern times.	**.715**	.303	.102
8. Internet-based therapy programs are as effective as conventional face-to-face psychotherapy.	**.714**	.643	.150
3. Internet-based therapy is better compatible with work and private life than conventional face-to-face therapy.	**.701**	.305	+++
13. I would prefer an Internet-based therapy to a conventional psychotherapy.	,414	**.820**	.393
11. Internet-based therapies are an appropriate alternative to conventional face-to-face psychotherapy.	.642	**.746**	.309
10. Regarding therapeutic success, it makes no difference whether contacts with a therapist are provided via the Internet or face-to-face in a psychotherapeutic practice.	.686	**.716**	.235
4. It makes no difference to me whether psychotherapy is conducted through the Internet or in a practice in a clinic.	.228	**.716**	.382
2. Internet-based therapies will replace conventional face-to-face psychotherapy in the future.	.324	**.614**	.390
5. Internet-based therapies will reach more people with mental health problems.	.312	.540	**.667**
6. Internet-based therapies can help bridging waiting time for conventional psychotherapy.	.480	.471	**.569**
14. Internet-based therapies will reach more patients and help them.	+++	+++	**-.110**

Extraction method: principal axis factor analysis; rotation method: promax with Kaiser normalization.
[a] Factor loadings smaller than .1 were suppressed (+++).
[b] Item rotation converged in 6 iterations.
[c] Mapping of items to factor: bold values indicate that the highest factor loading on a factor.
