# Supplementary material for: Current Views and Perspectives on E-Mental Health: An Exploratory Survey Study for Understanding Public Attitudes Toward Internet-Based Psychotherapy in Germany
Source: JMIR Ment Health. 2017 Feb 23;4(1):e8. doi: 10.2196/mental.6375 (PMC5378055; doi:10.2196/mental.6375)
Supplement: Multimedia Appendix 3 [file mental_v4i1e8_app3.pdf]

Multimedia Appendix 3

Table A3. Mapping of items of the e-therapy attitudes measure to constructs in technology acceptance model (TAM) framework.

Items of the e-attitudes measure	EFA-factor [a]	Related UTAUT constructs [b, c]
1. Internet-based therapies are modern and in line with our modern times.	**1 usefulness or helpfulness**	**Performance expectancy**<br>Perceived usefulness<br>**Social influence**<br>Subjective norm
2. Internet-based therapies will replace conventional face-to-face psychotherapy in the future.	**2 advantage or comparability**	**Performance expectancy**<br>Relative advantage<br>**Social influence**<br>Subjective norm
3. Internet-based therapy is better compatible with work and private life than conventional face-to-face therapy.	**1 usefulness or helpfulness**	**Performance expectancy**<br>Perceived usefulness<br>**Effort expectancy**
4. It makes no difference to me whether psychotherapy is conducted through the Internet or in a practice in a clinic.	**2 advantage or comparability**	**Performance expectancy**<br>Relative advantage<br>Outcome expectancy
5. Internet-based therapies will reach more people with mental health problems.	**3 e-accessibility (healthcare)**	**Performance expectancy**<br>Perceived usefulness<br>Relative advantage<br>**Social influence**<br>Subjective norm
6. Internet-based therapies can help bridging waiting time for conventional psychotherapy.	**3 e-accessibility (healthcare)**	**Performance expectancy**<br>Perceived usefulness<br>Relative advantage<br>**Social influence**<br>Subjective norm
7. Health insurance companies should cover the costs for Internet-based therapies.	**1 usefulness or helpfulness**	**Performance expectancy**<br>Perceived usefulness<br>**Social influence**<br>Subjective norm
8. Internet-based therapy programs are as effective as conventional face-to-face psychotherapy.	**1 usefulness or helpfulness**	**Performance expectancy**<br>Perceived usefulness<br>Outcome expectancy<br>Relative advantage
9. Trust in a therapist can be just as easily built on the Internet as in conventional face-to-face psychotherapy	**1 usefulness or helpfulness**	**Performance expectancy**<br>Perceived usefulness<br>**Facilitating factors**<br>Human support
10. Regarding therapeutic success, it makes no difference whether contacts with a therapist are provided via the Internet or face-to-face in a psychotherapeutic practice.	**1 usefulness or helpfulness**	**Performance expectancy**<br>Perceived usefulness<br>**Facilitating factors**<br>Human support
11. Internet-based therapies are an appropriate alternative to conventional face-to-face psychotherapy.	**2 advantage or comparability**	**Performance expectancy**<br>Relative advantage<br>Outcome expectancy
12. In case of mental health problems, I would attend an Internet-based therapy.	**1 usefulness or helpfulness**	**Performance expectancy**<br>Perceived usefulness<br>**Behavioral intention to use**
13. I would prefer an Internet-based therapy to a conventional psychotherapy.	**2 advantage or comparability**	**Performance expectancy**<br>Relative advantage<br>Outcome expectancy
14. Internet-based therapies will reach more patients and help them.	**3 e-accessibility (healthcare)**	**Performance expectancy**<br>Perceived usefulness<br>Relative advantage<br>**Social influence**<br>Subjective norm

[a] EFA = exploratory factor analysis.

[b]: UTAUT = unified theory of acceptance and use of technology (Venkatesh et al., 2003).

[c]: Bold terms indicate the dimensions of the UTAUT that were mapped to the content of the factors identified through the exploratory factor analysis for the e-therapy attitudes measure. The mapping was conducted qualitatively (face validity).
