# Supplementary material for: Current Views and Perspectives on E-Mental Health: An Exploratory Survey Study for Understanding Public Attitudes Toward Internet-Based Psychotherapy in Germany
Source: JMIR Ment Health. 2017 Feb 23;4(1):e8. doi: 10.2196/mental.6375 (PMC5378055; doi:10.2196/mental.6375)
Supplement: Multimedia Appendix 4 [file mental_v4i1e8_app4.pdf]

Multimedia Appendix 4

Table A4. Pattern Matrix: factor loadings of the exploratory factor analysis (EFA) with promax for the e-therapy attitude measure (N = 1558).

Items of the e-therapy attitudes measure	Factors [a-d] 1.Usefulness or Helpfulness	2.Relative advantage or Comparability
7. Health insurance companies should cover the costs for Internet-based therapies.	**.880**	-.128
1. Internet-based therapies are modern and in line with our modern times.	**.850**	-.256
3. Internet-based therapy is better compatible with work and private life than conventional face-to-face therapy.	**.828**	-.242
12. In case of mental health problems, I would attend an Internet-based therapy.	**.669**	.109
9. Trust in a therapist can be just as easily built on the Internet as in conventional face-to-face psychotherapy	**.629**	.205
8. Internet-based therapy programs are as effective as conventional face-to-face psychotherapy.	**.607**	.216
10. Regarding therapeutic success, it makes no difference whether contacts with a therapist are provided via the Internet or face-to-face in a psychotherapeutic practice.	**.497**	.374
4. It makes no difference to me whether psychotherapy is conducted through the Internet or in a practice in a clinic.	-.221	**.872**
13. I would prefer an Internet-based therapy to a conventional psychotherapy.	+++	**.833**
2. Internet-based therapies will replace conventional face-to-face psychotherapy in the future.	+++	**.645**
5. Internet-based therapies will reach more people with mental health problems.	+++	**.604**
11. Internet-based therapies are an appropriate alternative to conventional face-to-face psychotherapy.	.401	**.480**
6. Internet-based therapies can help bridging waiting time for conventional psychotherapy.	.284	**.348**
14. Internet-based therapies will reach more patients and help them.	+++	**-.106**

Extraction method: principal axis factor analysis; rotation method: promax with Kaiser normalization.

[a] Factor loadings smaller than .1 were suppressed (+++).

[b] Item rotation converged in 3 iterations.

[c] Mapping of items to factor: bold values indicate that the highest factor loading on a factor.

[d] The internal consistency for “relative advantage” was α = .78 and for the subscale “usefulnessor helpfulness” α = .89 (for the whole 14-item measure: α = .89). The cumulative explained variance amounted to 56.35 percent for the initial eigenvalue (factor 1: 43.3 %, factor 2: 13.01 %) and 49.9 % for the extracted sums of squared loadings (factor 1: 40.17 %, factor 2: 9.71 %).
